# Directed assembly of biofilm communities for marine biofouling prevention

**DOI:** 10.1128/aem.01392-25

**Published:** 2025-08-25

**Authors:** Cristina I. Amador, Naireen Fatima, Amanda Sofie Sejer Jakobsen, Lorrie Macario, Phillip Pichon, Nick Aldred, Mette Burmølle

**Affiliations:** 1 Department of Biology, University of Copenhagen 4321, Copenhagen, Denmark; 2 School of Life Sciences, University of Essex 2591, Colchester, United Kingdom; University of Delaware, Lewes, Delaware, USA

**Keywords:** multispecies biofilms, interspecies interactions, biofouling control, barnacle larvae settlement

## Abstract

Bio-based solutions depend on the application of living organisms to combat current challenges, including marine biofouling, which is characterized by the adhesion and growth of organisms on surfaces at sea. Such solutions traditionally involve single bacterial strains with specific, desirable activities or properties, thereby omitting the advantages conferred by the community context. We propose a novel approach, whereby desirable emergent properties of multispecies communities can be selected, such as those producing a thick and robust biofilm that is impenetrable to settling larvae. Here, bacterial biofilms from natural and artificial marine surfaces were studied, focusing on their adhesion, cohesion, stability, and antifouling properties both as single isolates and in multispecies communities. Using bottom-up assembly, we identified multispecies biofilm communities that exhibited greater tolerance to temperature variations compared to the component species. Additionally, some isolates, alone or as multispecies biofilms, prevented the settlement of barnacle larvae in short-term laboratory biofouling experiments. Broadly, our findings highlight the complexity of bacterial interactions within biofilms, revealing competition with occasional cooperation. More specifically, we present the possibility of a novel approach to biofouling control, whereby communities of marine isolates produce biofilms with the physical properties of a protective coating and, thus, move the industry a step toward environmentally friendly, regenerative antifouling coatings.

**IMPORTANCE:**

Marine biofouling poses a significant challenge to maritime industries, resulting in lower efficiency, higher maintenance costs, environmental impact and structural damage. Marine antifouling coatings are the first line of defense against biofouling and their biocidal mechanism of action has remained largely unchanged for decades. Although the concept of “living coatings” has been mooted previously, we take a novel approach. By exploiting useful emergent properties from multispecies communities, we propose that the resulting biofilms will be more environmentally stable than single-species biofilms, allow departure from a focus on active protection via toxic metabolites, and will eventually enable the development of biological coatings with desirable physical properties. By highlighting the competitive and cooperative dynamics within biofilms, the research identifies microbial communities that reduce barnacle larval settlement while tolerating environmental stressors like temperature variation. These findings are a first step towards eco-friendly, biofilm-based antifouling strategies that are both self-regenerating and environmentally compatible.

## INTRODUCTION

Aquatic microorganisms colonize submerged natural and artificial surfaces, leading to the formation of biofilms (1). In a biofilm, cells are attached to each other and/or to a surface, embedded in a matrix composed of extracellular polymeric substances (EPS) (2–4). Surface association is an ancient, universal survival strategy for microorganisms, offering advantages such as improved access to nutrients, enhanced interactions with other organisms, and greater resilience to environmental change (1). These features are of particular importance in aquatic environments where nutrients are often growth-limiting and ambient conditions are highly dynamic (5–7).

Biofilm formation in the ocean involves adhesion of different organisms, including bacteria, fungi, diatoms, protozoans, invertebrate larvae, and algal spores (8). However, bacteria are the most important microbes on marine surfaces and, by being early colonizers, they influence the structure and function of the mature biofilm (1, 9). While bacteria are often the initial colonizers of submerged surfaces, microalgae such as diatoms can also rapidly adhere, occasionally preceding bacterial attachment depending on specific environmental conditions. Bacteria dominate marine biofilms, with observed ratios of bacteria:diatoms:flagellates of 640:4:1, depending on the location (e.g., White Sea, [10]). Due to this intrinsic diversity, interspecies interactions play an essential role in biofilm stability, structure, and functionality. Interspecies interactions between bacteria can lead to emergent properties in multispecies biofilms including enhanced biomass, increased protection from toxic compounds, bacterial invasion, protozoan grazing and enriched metabolic capabilities (11–15). By identifying the most connected microbial taxa in such communities, functionally important microbes can be identified. These keystone taxa disproportionately affect microbial community structure and function (16). Consequently, surface- and biofilm-associated communities may thrive in extreme or hostile conditions that challenge the survival, growth and activity of individual microorganisms (17, 18).

The characteristics that promote bacterial survival in biofilms also present significant challenges for the maritime industry in the form of biofouling—the unwanted accumulation of organisms on manmade surfaces such as ship hulls, pipelines and aquaculture equipment (19, 20). Biofouling leads to increased drag, fuel consumption and maintenance costs, as well as posing risks to the structural integrity of marine infrastructure (21–24). However, the presence and composition of biofilms can also influence the structure of natural benthic communities by affecting recruitment, survival and growth rates of invertebrate populations (25, 26). Many sessile marine invertebrates—also referred to as macrofoulers—have evolved to use bacteria or their metabolites as indicators of specific ecological niches, suitable for settlement (27–30). The specific effects of bacteria on larval settlement are well-studied and, in some cases, sophisticated interkingdom signaling processes influence larval settlement either positively or negatively (31). Such relationships are, by definition, specific to single invertebrate and bacterial species, and the relationships between complex biofilms and macrofoulers remain unclear. Marine biofilms are intrinsically diverse, so generalizing mechanisms and harnessing them for broad-spectrum biofouling control would be challenging, e.g., for a ship hull that may be exposed to hundreds of different invertebrate species on a single voyage.

Traditionally, two routes have been considered toward innovation of this basic knowledge into fouling control. First, the active metabolites of bacteria could be purified or synthesized and incorporated into synthetic coatings. However, for the reasons outlined above and additional practical challenges in coating formulation, progress here has been limited. Alternatively, the bacteria themselves could be applied to a surface and allowed to release the active metabolites that control larval settlement. Still, these single bacterial species may not survive in field conditions or become augmented by other taxa quite rapidly, losing any antifouling effect the biofilm may have had to begin with. We propose a fundamentally different approach whereby active mechanisms are avoided, the “design” of the biofilm is blind to the taxonomic composition and the intrinsic, emergent properties of specific communities underpin the biofilm’s antifouling efficacy. In this paradigm, the biofilm functions as the coating, providing a protective physical barrier against larval settlement without the need for active mechanisms (e.g., toxicity).

This study investigated the characteristics of biofilms formed by bacteria, isolated from both artificial and natural marine surfaces, as single isolates and within multispecies communities, for their potential as “living coatings”. We aimed to identify properties relevant to biofouling control such as strong adhesion, cohesion, resilience, and antifouling effects. Additionally, we explored bacterial interactions to pinpoint specific combinations of taxa that may be desirable for enhanced biofilm productivity or, in contrast, have deleterious effects. It is known that enhanced biofilm formation, resistance to antimicrobials, and invasion can arise from synergistic interactions between marine epiphytic bacteria in multispecies biofilms (11).

In this study, we broadened our scope to include marine bacteria from two different milieus: epiphytic bacteria naturally associated with the alga *Ulva australis* (11) and bacteria collected from two marine vessel hulls in Helsingør (Denmark). We applied a bottom-up assembly approach by the source of isolation (alga or vessel) and identified multispecies communities with induced biofilm biomass compared to single isolates. Using this strategy, we identified biofilm communities that reduced the settlement of cyprid larvae of the globally distributed barnacle *Amphibalanus improvisus* (32) in laboratory assays. We also showed that bacterial interactions in multispecies biofilms are complex and have a dominant competitive nature but also that cooperative interactions are dependent on the specific taxa or species involved. By understanding and harnessing such interactions, it may be possible to design biofilm-based approaches that offer self-regenerating, eco-friendly protection for underwater surfaces. We therefore consider that our results are a first step toward the development of biotechnological strategies for biofouling management.

## MATERIALS AND METHODS

### Sampling and isolation

**Fig 1:**
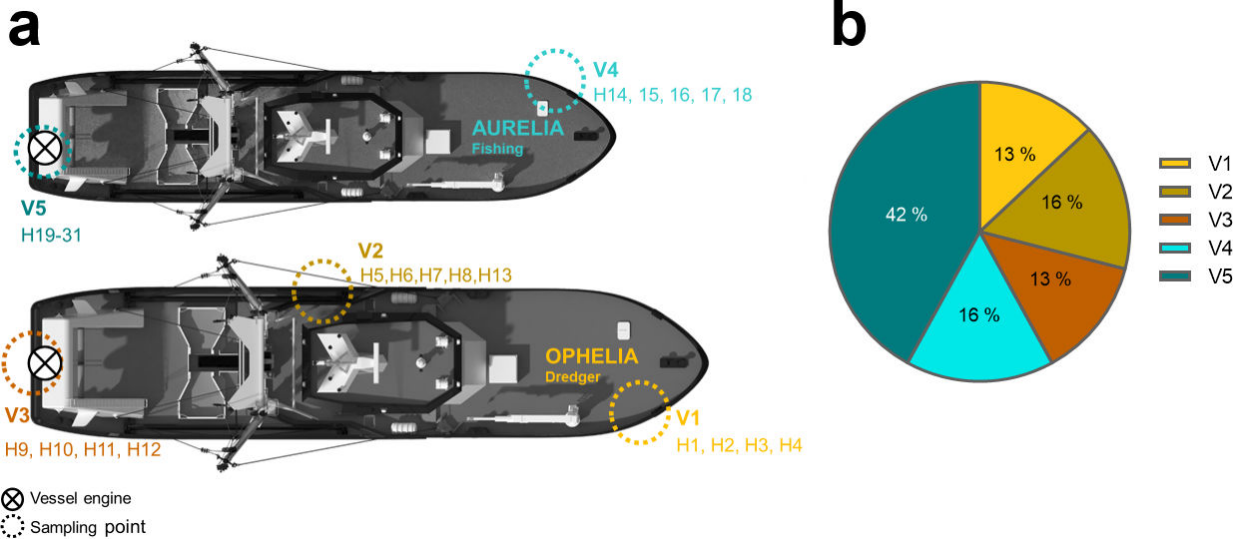
Sampling from the submerged hulls of Aurelia and Ophelia vessels. (**a**) Sampling points of the Ophelia and Aurelia (top) and Ophelia (bottom) vessel hulls. Each isolation point is marked by dotted circles (◌, V1–V5) and the strains derived from it indicated as H#. The vessel propeller is indicated as a crossed circle (⊗). (**b**) The distribution of isolates is represented in a pie chart divided by sampling points.

Biofilm samples were collected on 6 October 2020 from the submerged hulls of Aurelia and Ophelia vessels in Helsingør harbor (Denmark). Samples were collected from five sampling points, two from Aurelia and three from Ophelia (Fig. 1a; Table S1), including sampling points close to the vessels’ propellers. Each sample was taken using a sterilized brush attached to a telescopic stick. The brushes were immediately soaked in 15 mL 1 x nine-salt solution (NSS) in 50 mL Falcon tubes (33). Three water samples were also taken, two from V1 and V2 locations (next to Ophelia vessel, 56° 02' 29.9" N, 012° 36' 47.3" E) and a third one 25 meters away from Aurelia next to a dock, location 56°02'31.1"N 12°36'48.3"E. Water temperature while sampling was 13.5°C and air temperature 14.5°C.

The samples were vortexed for 10 min to detach the collected material from the brush. Then, a dilution series 10^−1^–10^−8^ was prepared in 1 x NSS for each sample, and 100 µL was plated out in duplicates on Väätanen nine-salt solution (VNSS) marine agar medium (33) supplemented with 40 µg/mL Congo red and 20 µg/mL Coomassie brilliant blue to aid in identification of different colony morphology. Cycloheximide (40 µg/mL) was added to the agar plates to prevent fungal growth (34). Glycerol stocks (15%) were prepared from each sampling point, using a 2 mL sample and stored at −80°C. The rest of the water samples were stored at −20°C for metagenomics sequencing. Each sample set was incubated at 18°C or 24°C for 4–5 days. After incubation, 31 colonies were selected based on distinct colony morphology and further re-streaked on the same media in order to retrieve pure isolates. Isolated colonies were used to inoculate 5 mL liquid VNSS cultures and incubated for 1–2 days at 24°C and 250 rpm. These 31 isolates were frozen as pure isolates using 15% glycerol (final concentration), stored at −80°C, and referred to as vessel isolates.

### Bacterial strains and growth conditions

Besides the 31 vessel isolates, 15 marine bacteria isolated from the alga *Ulva australis,* formerly known as *Ulva lactuca* (35), were also used in this study and referred to as alga isolates. All strains used in the study are listed in Table 1.

**TABLE 1:** Bacterial strains used in this study

Isolate	Description	16S ID^*a*^	Source
H01	Vessel isolate	*Marinobacter adherens*	This study
H02	Vessel isolate	*Alteromonas oceani* strain S35	This study
H03	Vessel isolate	*Marinobacter adhaerens HP15*	This study
H04	Vessel isolate	*Cellulophaga baltica strain NN015840*	This study
H05	Vessel isolate	*Pseudoalteromonas mariniglutinosa KMM 3635*	This study
H06	Vessel isolate	*Pseudoalteromonas prydzensis MB8-11*	This study
H07	Vessel isolate	*Cellulophaga tyrosinoxydans EM41*	This study
H08	Vessel isolate	*Pseudoalteromonas neustonica PAMC 28425*	This study
H09	Vessel isolate	*Pseudoalteromonas prydzensis MB8-11*	This study
H10	Vessel isolate	*Dokdonia diaphoros strain MSKK-32*	This study
H11	Vessel isolate	*Pseudoalteromonas sp*.	This study
H12	Vessel isolate	*Pseudoalteromonas mariniglutinosa KMM 3635*	This study
H13	Vessel isolate	*Marinobacter adhaerens HP15*	This study
H14	Vessel isolate	*Cellulophaga baltica NN015840*	This study
H15	Vessel isolate	*Paraglaciecola hydrolytica S66*	This study
H16	Vessel isolate	*Paraglaciecola hydrolytica S66*	This study
H17	Vessel isolate	*Microbacterium foliorum P 333/02*	This study
H18	Vessel isolate	*Microbacterium foliorum P 333/02*	This study
H19	Vessel isolate	*Pseudoalteromonas prydzensis MB8-11*	This study
H20	Vessel isolate	*Pseudoalteromonas tetraodonis GFC IAM 14160*	This study
H21	Vessel isolate	*Leeuwenhoekiella aequorea R7695*	This study
H22	Vessel isolate	*Maribacter dokdonensis DSW-8*	This study
H23	Vessel isolate	*Pseudoalteromonas neustonica PAMC 28425*	This study
H24	Vessel isolate	*Erythrobacter vulgaris 022 2-10*	This study
H25	Vessel isolate	*Psychrobacter piscatorii T-3-2*	This study
H26	Vessel isolate	*Psychrobacter piscatorii T-3-2*	This study
H27	Vessel isolate	*Psychrobacter piscatorii T-3-2*	This study
H28	Vessel isolate	*Dokdonia diaphoros MSKK-32*	This study
H29	Vessel isolate	*Psychrobacter piscatorii T-3-2*	This study
H30	Vessel isolate	*Psychrobacter piscatorii T-3-2*	This study
H31	Vessel isolate	*Pseudoalteromonas prydzensis MB8-11*	This study
621	Alga isolate	*Microbacterium phyllosphaerae* (Isolate 2.04)	(11)
622	Alga isolate	*Phaeobacter gallaeciensis* (Isolate 2.10)	(35)
623	Alga isolate	*Shewanella japonica* (Isolate 2.12)	(11)
624	Alga isolate	*Pseudoalteromonas gracilis* (Isolate 2.14)	(35)
625	Alga isolate	*Pseudoalteromonas marina* (Isolate 2.2)	(11)
626	Alga isolate	*Alteromonas sp.* (Isolate 2.19)	(35)
627	Alga isolate	*Dokdonia donghaensis* (Isolate 2.3)	(11)
628	Alga isolate	*Acinetobacter Iwoffii* (Isolate 2.34)	(11)
629	Alga isolate	*Dokdonia donghaensis* (Isolate 2.6)	(11)
630	Alga isolate	*Bacillus paramycoides* (Isolate 2.40)	(11)
631	Alga isolate	*Pseudoalteromonas spiralis* (Isolate 16)	(11)
632	Alga isolate	*Enterovibrio calviensis* (Isolate 22)	(11)
633	Alga isolate	*Cellulophaga baltica* (Isolate 26)	(11)
634	Alga isolate	*Cellulophaga baltica* (Isolate 36)	(11)
635	Alga isolate	*Cellulophaga algicola* (Isolate 42)	(11)

^*a*^ Isolate identification by 16S rRNA gene analysis. The sequences had >99% base identity to the closest relative in GenBank.

All marine strains were cultured on VNSS medium without starch or on Marine Broth 2216 (MB, Sigma-Aldrich) at 24°C and 250 rpm, unless otherwise specified. When solid media were needed, 1.5% bacteriological agar (wt/vol, VWR) was added.

### 16S rRNA Sanger sequencing

DNA was extracted from each isolate using the DNeasy Blood & Tissue kit (Qiagen) with a few modifications. Briefly, 1 mL cultures were spun for 2 minutes at 13,000 rpm and lysed with ATL buffer, followed by proteinase treatment. DNA was quantified using the Qubit dsRNA HS kit. Hypervariable regions V1–V9 of the 16S rRNA gene were amplified through PCR using forward primer 27 f (5′ AGAGTTTGATCMTGGCTC 3′) and reverse primer 1492 r (5′ AGCGYTACCTTGTTACCACTT 3′) with PCRBIO HiFi polymerase (PCR Biosystems; 3 mM MgCl_2_, 1 mM dNTPs, 0.4 µM primers, 1 U PCRBIO HiFi and 50 ng genomic DNA). Cycling conditions were 95°C for 2 minutes; 30 x (95°C for 10 seconds; 55°C for 15 seconds and 72°C for 1 minute); final extension 72°C for 5 minutes.

### 16S rRNA gene amplicon sequencing of water and biofilm samples

Hypervariable regions V3–V4 of the 16S rRNA gene were amplified through PCR using forward primer 341 f (5′-CCTAYGGGRBGCASCAG-3′) and reverse primer 806 r (5′-GGACTACHVGGGTWTCTAAT-3′) (36–38). Temperature settings for one cycle were: 95°C for 15 s; 56°C for 15 s; 72°C for 30 s. The primers were barcoded so each sample could be uniquely identified by post-sequencing in the second PCR. Negative controls were included for the extraction and PCR amplification procedures. In each case, the negative controls yielded no amplification, confirming the absence of contamination. All final PCR products were purified using HighPrep PCR (MAGBIO, USA), based on the paramagnetic bead technology and normalized using the SequalPrep Normalization plate kit (Invitrogen, USA). Further cleaning and concentration were done by using the DNA Clean & Concentrator−5 Kit (Zymo Research, Irvine, CA, USA). Concentrations were then determined using the Quant-iT High-Sensitivity DNA Assay Kit (Life Technologies). The final library was loaded at 9 pM on the Illumina MiSeq instrument using V3 chemistry (2 × 300 cycles, Illumina San Diego, CA).

### Alpha and beta diversity analysis

Amplicon sequencing data analysis was conducted in R (2022.02.1). Initial preprocessing of the ASV table was conducted using the phyloseq package (v1.40.0) (39). ASVs were normalized to generate a relative abundance of taxa present in each sample. All downstream analyses were performed on this normalized ASV table unless mentioned.

The microbial community composition was compared across sample groups (Ophelia, Aurelia, and water samples). Alpha and beta diversities were calculated using the phyloseq package v1.40.0 and visualized with ggplot2 v2.2 (40) in R v3.4.1. We used observed richness, Chao1, Shannon, and Simpson as alpha diversity indices. Furthermore, the beta diversity was calculated using unweighted UniFrac distances (phyloseq package), which account for the phylogenetic relationships and the presence/absence of taxa and visualized by principal coordinates analysis (PCoA) for microbiome analysis. These distances were subsequently analyzed using permutational multivariate analysis of variance (PERMANOVA, Vegan package) to test for differences in the community composition among sample groups.

### Growth and biofilm biomass screening of marine isolates

All marine isolates were assessed for endpoint planktonic growth and biofilm formation in monocultures at 24°C and 120 rpm (orbital shaking) in VNSS and MB after 24 and 48 hours (h) of incubation. First, strains were inoculated in 5 mL VNSS or MB medium and incubated for at least 18 h at 24°C and 250 rpm. Aliquots of 2 mL of these overnight cultures were washed thrice with 1 x NSS and resuspended in 2 mL VNSS or MB medium, respectively. Washed cultures were diluted 100 x in VNSS or MB medium and 150 µL inoculated in microtiter plates with six technical replicates per isolate, using non-inoculated media as blank controls. Replicate plates were prepared for 24 and 48 h incubation for each medium.

After incubation, microtiter plates were read for optical density at 600 nm (OD_600_) using a BioTek ELx808 plate reader. OD_600_ was used as a measure of endpoint planktonic growth (carrying capacity or growth yield). Biofilm formation was evaluated by a modified version of the crystal violet (CV) assay where the bacteria are grown in microtiter plates (41) for 24 and 48 h. Biofilm biomass was quantified as the absorbance of CV at 590 nm (A_590_).

To select media and incubation temperatures for community assembly, mean biofilm biomass (mean A_590_) and planktonic growth (yield, mean OD_600_) were calculated per population (vessel or alga origin) and used as a threshold (see Table S2). Additionally, we calculated the 75th percentile of biofilm biomass (or third quartile) and planktonic growth to identify top 25% biofilm or growth-performing isolates (see Table S3).

### Community assembly

After initial isolate screening, assembly was performed with either alga or vessel isolates, starting with co-cultures and focusing on isolates ranking within the top 25%. Each combination contained at least one isolate that demonstrated high adhesion capability in the monoculture screening. For co-culture inoculation, we followed the same procedure as for single isolates but mixing a 1:1 vol ratio of 100 x diluted cultures. We tested a total of 79 co-culture combinations (Table S4), 70 from vessel isolates and nine from alga isolates, with ≥3 biological replicates as independent experiments and six technical replicates per combination. The predicted co-culture biofilm biomass was calculated as the average of the two species in each combination. Combinations showing biofilm induction >1.5 fold in ≥2 biological replicates compared to the predicted co-culture biofilm were chosen for triple cultures (multispecies).

A total of 90 multispecies (three species) combinations were assembled, 10 with alga isolates and 80 with vessel isolates. Mixtures with a 1:1:1 vol ratio of 100 x diluted isolate cultures (Table S5) were prepared, and 150 µL was inoculated in microtiter plates as six technical replicates and three biological replicates in independent experiments. Biofilm quantification was performed as described in the previous section. The predicted triple-culture biofilm biomass was calculated as the average of the three species in each combination. Combinations showing biofilm induction >1.5 fold in ≥2 biological replicates, compared to the predicted triple-culture biofilm, were chosen for temperature tolerance assays.

### Temperature resilience assays

A total of eight triple-species communities, namely, TV3, TV 27, TV31, TV32, TA1, TA4, TA5, and TA7 (TV = triple species vessel; TA = triple species alga, Table S3), were tested for temperature resilience. As described above, overnight cultures were washed thrice in 1 x NSS and 100 x diluted in the VNSS medium. Then, triple-species mixtures were prepared using 1:1:1 vol ratios of each species, and 150 µL was inoculated in microtiter wells with eight technical replicates and ≥5 biological replicates as independent experiments. Each plate included monoculture controls for all species used. Plates were incubated at 4°C, 10°C, or 24°C for 48 and 72 h with 120 rpm orbital shaking. Planktonic growth (OD_600_) and biofilm biomass (A_590_) were then measured. Average biomass values were calculated for each community, temperature, and incubation time. Additionally, average biomass was calculated per temperature and incubation time to serve as the reference for induced/reduced biofilm formation compared to the population mean.

### Barnacle cyprid larvae settlement assays

Barnacle larvae (cyprids) of *A. improvisus* were produced using laboratory-maintained adult barnacles as broodstock. These periodically release stage 1 nauplius larvae. Nauplii were cultured on a diet of *Thalassiosira pseudonana* and *Skeletonema costatum* for around 1 week before metamorphosing into cyprids. Cyprids were used in settlement assays when 3 days old, with prior storage at 6°C.

Single-species bacterial cultures of H02, H13, H22, H25, H26, H27, and H29 were maintained in full-strength marine broth (DIFCO MB) and sub-cultured weekly. Prior to a barnacle assay, bacterial sub-cultures of the required volume were prepared and grown for 48 h. Optical density measurements (OD_600_) were taken to ascertain the relative cell density and starting volumes of culture (for multi-species biofilms) were adjusted to ensure approximately equal amounts of each strain. PVC coverslips were added to the growth medium containing the desired species in the correct ratios. These were incubated at 28°C for 48 h. After this time, the liquid medium was discarded and a 0.5 mL droplet of artificial seawater (22ppt TropicMarin Reef Salt in reverse osmosis water) was added to each coverslip. Barnacle cyprid larvae were then added, 10 per coverslip, incubated in the dark at 28°C for 48 h and then observed for settlement. Settlement percentage (e.g., Fig. 7) was calculated by expressing those settled as a proportion of the total initially added and averaging these values over six replicates. Cyprid settlement was tested on i) biofilms of single marine isolates and ii) biofilms of a community composed of H02, H22, and H25 isolates on PVC coverslips, using non-inoculated coverslips as a standard surface to confirm that cyprid settlement occurred within the normal range. Similarly, settlement assays were conducted using effluent controls; that is, planktonic cultures of marine isolates instead of biofilms to identify the effects of bacterial metabolites. In these assays, the growth medium containing the desired species in the correct ratios was added up to 1 mL in the wells of a polystyrene 24-well microtiter plate (Costar, Corning). An additional 1 mL of the marine broth was also added. These well-plate cultures were incubated at 28°C for 48 h, after which the liquid medium was removed and replaced with seawater for 48 h. This seawater medium was then transferred to clean wells, and the settlement assay was conducted as above using 10 larvae per well.

### Assessment of biofilm morphology

Epifluorescence microscopy was performed to visualize the morphology and surface coverage of biofilms formed by single bacterial isolates (H02, H13, H22, H25, H26, H27, and H29) and a multispecies combination (H02 +H22+H25). Biofilms were grown on UV-sterilized PVC coverslips under the same conditions as the larval settlement assays. Briefly, overnight cultures of each isolate were grown in DIFCO MB at 24°C for 18 h and then diluted to an initial OD₆₀₀ of 0.1 in fresh DIFCO MB. For the multispecies treatment, equal volumes of OD₆₀₀ 0.1 cultures of H02, H22, and H25 were mixed to achieve an approximately equal representation of each strain. A volume of 5 mL of either single-species or multispecies culture was added to 12-well plates containing sterile PVC coverslips and incubated for 48 h at 28°C to allow biofilm formation.

Following incubation, PVC coverslips were gently rinsed with 1 x PBS to remove planktonic or loosely attached cells and stained with 25 µL of a 5 µM SYTO9 solution. PVC coverslips, containing the biofilms, were then mounted on standard glass slides using Mowiol mounting medium applied to the edges to secure the PVC surface and incubated in the dark for 30 minutes. Imaging was conducted using a Zeiss Axio Observer Z1 epifluorescence microscope equipped with LD Plan-NEOFLUAR 20 x and N-ACHROPLAN 63 x objectives, with numerical apertures of 0.4 and 0.85, respectively. Both low- (20 x) and high-(63 x) magnification images were captured to document the overall biofilm architecture and fine structural features, respectively.

For each biofilm condition, three biological replicates were prepared and imaged. The images presented are representative of consistent morphologies observed across replicates.

### Statistical analysis

All analyses and plotting were done in RStudio v4.1.1 and package “ggplot2” v3.4.2. All data sets were tested for normality and equal variance using the Shapiro-Wilk (base R) and Levene’s (package “car” v3.1.2) tests, respectively. Data were not normally distributed, and therefore a Kruskal-Wallis test (base R) was used when comparing more than two groups, unless otherwise specified. When comparing only two groups, a Wilcoxon's rank *t*-test was applied, usually comparing biofilm biomass based on a specific factor. A *P*-value of 0.05 was used as the threshold for statistical significance (*P* < 0.05).

For temperature resilience assays, a Kruskal-Wallis test was applied individually to assess the influence of temperature (Biofilm ~ Temperature), while a Wilcoxon’s rank test was utilized to assess the effect of time (48 vs 72 hours) and culture condition (monospecies vs multispecies). We also tested the impact of time and culture within temperature (4°C, 10°C, and 24°C), using a Wilcoxon’s rank *t*-test. For example, 24°C: Biofilm ~ Time or Biofilm ~ Culture. All comparisons are available in Table S6.

To assess significant induction of biofilm biomass between multispecies and monospecies communities at the different temperatures or incubation time points, we applied a Wilcoxon's rank *t*-test comparing a multispecies mixture with the best monospecies in such mixture. All data can be found in Table S7.

To assess significant differences in cyprid settlement, Dunnett’s multiple comparison test was used on comparisons between each treatment group (isolate biofilm or effluent) and the control group (PVC), with *P* < 0.05. When comparing settlement in the biofilm community and its control (PVC), the Holm-Sidak method was applied, with *P* < 0.05.

## RESULTS

### Sampling and identification of vessel-adhering bacteria

Sampling from Aurelia and Ophelia vessels resulted in 31 bacterial isolates that were further subjected to 16S rRNA gene sequencing for identification. Forty-two percent of all isolates were derived from Aurelia’s propeller sampling point (V5, Fig. 1; Table S1). All isolates matched previously sequenced marine bacterial species/genera (Table 1).

**Fig 2:**
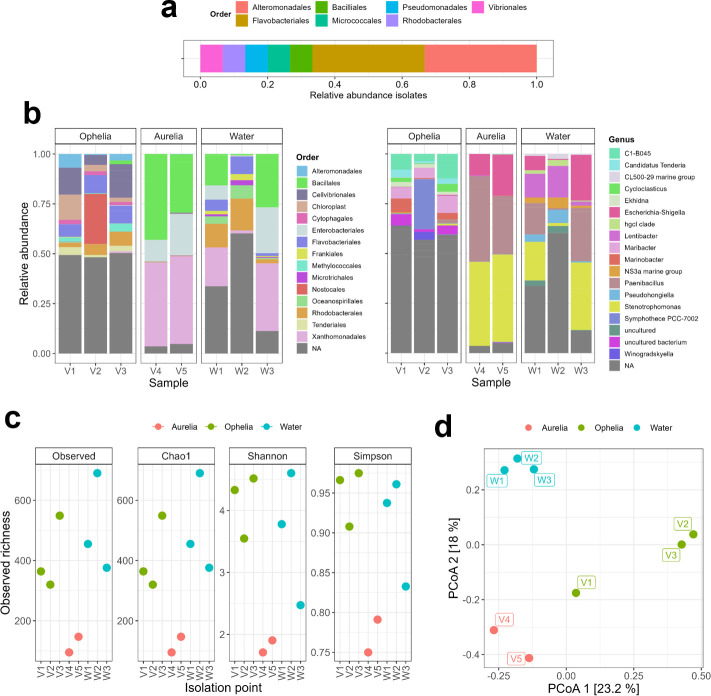
Taxonomic identity and diversity of vessel and water samples. (**a**) Taxa identity from Aurelia and Ophelia vessel isolates, based on 16S rRNA gene sequencing. The count indicates the number of isolates belonging to each taxonomical order. (**b**) Taxonomic composition of the samples depending on the isolation point: Ophelia vessel (V1–V3), Aurelia vessel (V4–V5), and marine water samples collected near the vessels (W1–W3). (**c**) Alpha diversity of the samples per isolation point, measured by different coefficients: observed richness, Chao1, Shannon, and Simpson, from left to right. (**d**) Principal component analysis of beta diversity for vessel and water samples (unweighted UniFrac).

A third of the isolates matched with species of the *Alteromonas* and *Pseudoalteromonas* genera (Table 1; Fig. 2a), frequently isolated from marine waters and associated with marine invertebrates, algae, plants, and animals (42). They generally grow fast with minimal nutritional requirements (43) and produce bioactive compounds with antibacterial, antifungal, algicidal, and antifouling properties, as well as a broad profile of enzymatic activity (44–47). Moreover, *Alteromonas* spp. are early colonizers of copper-based antifouling paint, while Pseudoalteromonads are prolific biofilm formers and may repel or induce settlement of other bacteria or bivalve larvae (48–50).

Isolates H4, H7, and H14 matched the *Cellulophaga* genus (*Bacteroidetes*). *Cellulophaga* spp. have been isolated from a variety of coastline biotopes (51, 52), including marine biofilms (53) and have potential biotechnological properties, including antifouling and algicidal activities, or the production of marine polysaccharide-degrading enzymes, such as carrageenase, agarase, and cellulase (54–58).

The closest relatives of isolates H25, H26, H27, H29, and H30 were *Psychrobacter piscatorii* (59). *Psychrobacter* species are endemic in extremely cold and saline environments with adaptations to significant temperature variations. They are only marginally successful in warmer habitats, however (60, 61). *Psychrobacter* enrichment has been reported in aquatic environments contaminated with hydrocarbons (62).

The closest matches to H13 and H22 were *Marinobacter adhaerens* (63) and *Maribacter dokdonensis*, respectively (64). *Marinobacter* species are ubiquitous and phenotypically versatile (65). Other genera identified were *Paraglaciecola* (66), *Dokdonia* (64), *Erythrobacter* (67), and *Leeuwenhoekiella* (68).

In general, Gram-negative bacteria dominated within the sampled isolates, reflecting the overall bacterial composition of marine habitats (69–71), while only two isolates, H17 and H18, were identified as Gram-positive bacteria (*Microbacterium foliorum* [72]).

### Community composition differed across vessel samples

To inform the design of laboratory-assembled communities, we assessed the composition and diversity of bacteria cultured and isolated from vessel biofilms and compared these to the broader microbial profiles of the original vessel and surrounding seawater samples (V1–V5; W1–W3). We conducted 16S rRNA amplicon sequencing of these samples to identify enriched or dominating taxa (Fig. 2b) and calculated alpha and beta diversity indices across all samples (Fig. 2c and d). While the Ophelia-derived communities exhibited greater diversity than those from Aurelia and some taxonomic differences were observed across sample points, our focus was not to characterize ecological variation *per se*, but rather to understand the cultivated microbial pool available for assembling synthetic communities. For V1 and V3 sampling points, bacterial orders *Cellvibrionales*, *Flavobacteriales*, and *Rhodobacterales* were the most abundant, while *Nostacales* dominated V2 (Fig. 2b). Aurelia communities were, however, composed of just *Bacillales*, *Enterobacteriales*, and *Xanthomonadales*. The water samples W1 and W2 showed different compositions compared to the vessel samples corresponding to the sampling points, indicating that the adhered bacterial communities differed from the planktonic community. However, the taxonomic composition of W3, sampled close to the Aurelia vessel was similar to the hull samples (V4 and V5), although with lower alpha diversity (Fig. 2b and c). Alpha diversity was significantly different for Shannon and Simpson coefficients between the three types of samples (*P* = 0.0474 and *P* = 0.0285, respectively). Moreover, multiple comparisons revealed significant differences in alpha diversity between Aurelia and Ophelia vessel communities, but not compared to the water samples (Shannon, *P* (Aurelia-Ophelia) =0.0441; Simpson, *P* (Aurelia-Ophelia) =0.02646). Beta diversity was also significantly different between the different sample types (*P* = 0.006, PERMANOVA) (Fig. 2d).

Overall, differences in community composition and diversity were observed between vessel and marine water samples, providingthe context for the culture isolates used in subsequent laboratory-based biofilm assembly.

### Biofilm screening revealed greater biofilm biomass of vessel isolates compared to algal isolates

**Fig 3:**
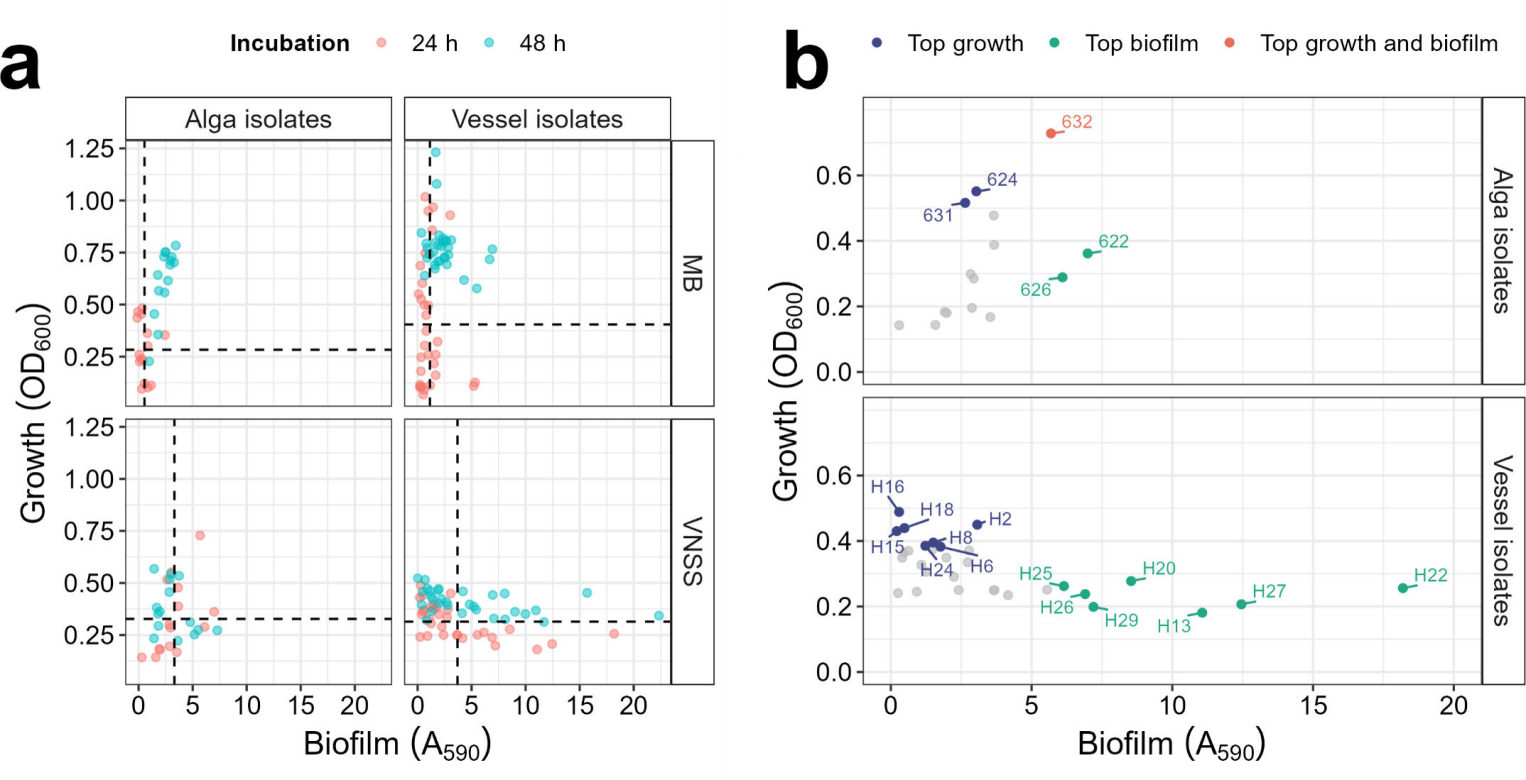
Screening of biofilm formation and planktonic growth of 46 marine isolates. (**a**) Biofilm formation (A_590_) and growth yield (OD_600_) of alga and vessel isolates in marine broth (MB) and VNSS media after 24 and 48 h incubation. Points represent mean values of six replicates. Dashed lines denote the 3rd quartile of the entire population for 24 h (red) and 48 h (blue). (**b**) Selected isolates from the screening (a) in VNSS after 24 h incubation, displaying biofilm formation (blue), growth (green) or both (red) above the third quartile. All other isolates are shown as grey points.

An initial screening for biofilm formation and growth was conducted using the 31 sampled vessel isolates and 15 epiphytic bacteria obtained from coastal algae (Table 1) (11, 35). This screening aimed to identify bacterial isolates exhibiting superior biofilm formation capacity and/or growth yields, suitable for use in subsequent community assembly. All 46 marine isolates were incubated individually in microtiter plates containing either VNSS or MB media (replicates of eight wells), and biofilm adhesion (crystal violet, A_590_) was measured after 24 and 48h (Fig. 3a).

Both media and incubation time impacted mean biofilm biomass for all isolates (Table S2). Specifically, VNSS significantly increased biofilm biomass compared to MB, irrespective of the isolation origin, but only at the 24 h sampling point (Vessel: 3.22-fold, *P* = 0.0012; Alga: 6.04, *P* = 3.51×10^−03^). Prolonged incubation only had a significant effect on biofilm biomass when MB was used as the growth medium, suggesting a growth and/or adhesion lag effect in this medium, or potentially due to higher nutrient concentrations in MB, supporting growth for a longer duration (Vessel: 2.09-fold, *P* = 1.12×10^−05^; Alga: 4.36, *P* = 8.60×10^−04^; 48 vs 24 h). When comparing vessel vs alga isolate populations, the former generally produced higher biofilm biomass than the latter, irrespective of the incubation time and media. This not only impacted mean biofilm biomass but especially the 75th percentile, utilized here as a threshold for identification of top-adhesive isolates (Table S2, descriptive statistics).

Based on these results, we selected VNSS and 24 h as the medium and incubation time, respectively, for community assembly. Strains exhibiting biofilm formation above the third quartile of each population (top 25%; 24 h incubation, VNSS) were selected for dual and triple strain combinations (Fig. 3b; Table S3). Among these strains, H13, H20, H22, H25, H26, H27 and H29 were chosen from the vessel isolates (Fig. 3b). Isolate H27 (*Psychrobacter piscatorii*) displayed the highest biofilm biomass, 12.4 (A_590_), 3.4-fold higher than the mean for all vessel isolates. Additionally, we selected isolates H02, H06, H08, H15, H16, H18, and H24 as they exhibited higher planktonic growth (culture yield) than 75% of the population. Alga isolates 622 and 626 were selected as good biofilm formers and 624 and 631 as isolates displaying high growth yields, while isolate 632 exhibited good biofilm and growth. These isolates had consistently shown high values for biofilm: planktonic ratio in monoculture in another study, where biofilm measurements were normalized by their planktonic growth (11).

### Biofilm reduction dominated in co-cultured marine communities

The isolates selected in the initial screening were subjected to a bottom-up community assembly approach with increasing complexity for each isolation origin (vessel isolates or alga isolates). Dual-species combinations with biofilm-inducing properties were selected for inclusion in triple-species mixtures, so while we increased the community complexity, we also reduced the number of mixed-species combinations. Seventy-nine dual-species combinations were grown and tested for biofilm induction, 70 including vessel isolates and nine for alga isolates (Table S4).

**Fig 4:**
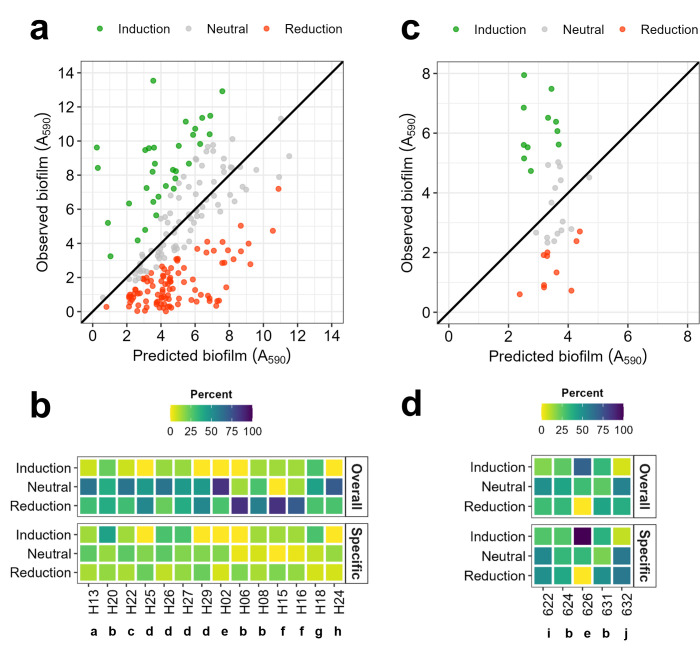
Effect of co-culture communities and their members on biofilm biomass. Biofilm formation (A_590_) was determined using a crystal violet assay after 24 h incubation in VNSS medium and 120 rpm orbital shaking. Top panels (**a and c**) represent biofilm induction/reduction of dual-species mixtures (y axis, Observed) compared to the average of their monospecies (x axis, Predicted). Each point represents one combination. The diagonal line indicates the threshold of induction (co-culture-monoculture ratio = 1); co-cultures above the line indicate induction and below reduction. Combinations showing biofilm production changes > 1.5-fold (compared to the average of their monospecies biofilms) are shown in colored points (induction: green, reduction: red) or else indicated in gray. (**a**) Co-culture of vessel isolates, n = 70 combinations, three biological replicates. (**c**) Co-culture of alga isolates, n = 9 combinations, four biological replicates. Bottom panels (**b and d**) are heatmaps illustrating how frequently specific isolates were present in co-cultures of induced, neutral, or reduced biofilm formation, respectively (shown in percentages). Dissimilar letters indicate different genera. a: *Marinobacter*, b: *Pseudoalteromonas*, c: *Maribacter*, d: *Psychrobacter*, e: *Alteromonas*, f: *Paraglaecicola*, g: *Microbacterium*, h: *Erythrobacter*, i: *Phaeobacter,* and j: *Enterovibrio*. Overall: overall presence frequency (percentage) of the strain per type of effect, calculated as the number combinations where the strain is present in a co-culture of a specific effect/ number of combinations of that species (in percentage). Specific: effect-specific presence of the strain, calculated as the number of combinations where the strain is present in a co-culture of a specific effect/ number of combinations with that effect (in percentage). The color scale indicates the percentage of strain related to presence in co-cultures with a specific outcome (induction, neutral, or reduction), where yellow color denotes less presence in biofilm-induced/ -reduced combinations, while dark blue indicates higher presence. (**b**) Vessel-isolate communities, n _reduction_ = 99, n _induction_ = 32, and n _neutral_ = 79. (**d**) Alga-isolate communities, *n* = 30, n _reduction_ = 10, n _induction_ = 11, and n _neutral_ = 15.

We calculated co-culture vs mono-culture biofilm ratios by comparing the biofilm biomass (A_590_) produced in co-culture to the predicted biomass, defined as the average biofilm biomass of the corresponding monocultures. To distinguish meaningful changes in biofilm formation, we defined an induction/reduction threshold of 1.5-fold. Co-cultures were considered to have a neutral effect when the biofilm ratio (co-culture/ predicted biomass) was between –1.5 and 1.5 (Fig. 4, gray points). Ratios above 1.5 indicated biofilm induction (Observed co-culture >Predicted; Fig. 4, green points), while ratios below –1.5 indicated biofilm reduction (Predicted >Observed co-culture; Fig. 4, red points).

For vessel co-culture communities, 47.1% resulted in biofilm reduction, while only 15.2% of the combinations led to an increase in biofilm biomass compared to the predicted co-culture biomass, calculated by the simple addition of the individual isolates (Fig. 4a). Several pairs induced biofilm biomass reproducibly, such as H27 +H18 (*Psychrobacter* and *Microbacterium*), H20 +H15 (*Pseudoalteromonas tetraodonis* and *Paraglaecicola*), and H13 +H27 (*Marinobacter* and *Psychrobacter*) and thereby constituted good candidates for testing in multispecies combinations. Furthermore, we analyzed how frequently each isolate was present in co-cultures of induced vs reduced biofilm formation. H20, H26, H27 and H18 (*Pseudoalteromonas tetraodonis, Psychrobacter piscatorii, and Microbacterium foliorum*) were present in higher numbers of biofilm-inducing communities (15%–29%), while H06, H08, H15 and H16 (*Pseudoalteromonas prydzensis, Pseudoalteromonas neustonica,* and *Paraglaecicola hydrolytica*) were often present in communities with reduced biofilm biomass (57%–86%, Fig. 4b). Given that some isolates were included in fewer combinations (top growth isolates, Table S4) we also calculated the relative frequency of each species, accounting for the number of combinations where that effect was observed. Here we observed equivalent, but less distinct, effects. Interestingly, no link between specific genera and overrepresentation in biofilm-inducing or reducing communities was identified, indicating that closely related isolates might possess distinct traits and impact community dynamics differently. Based on the above, we excluded H06 and H24 from the three-species combinations as they were never present in combinations leading to biofilm biomass induction. H02 was included in further co-culture experiments since it formed biofilm biomass close to the threshold defining top biofilm isolates. We excluded H20 due to its aggregative phenotype in liquid culture.

For co-cultures of alga isolates, the proportions of combinations causing biofilm reduction and induction were similar with 28% and 31%, respectively (Fig. 4c). In particular, the 626 + 631 co-culture (*Alteromonas* sp. and *Pseudoalteromonas spiralis*) reproducibly induced adhesion in all four biological replicates. Whenever included, biofilm biomass of combinations containing strain 626 was consistently induced regardless of its partner (Fig. 4d). This indicates that 626, *Alteromonas* sp., serves as an effective partner for inducing biofilm formation in the alga-derived combinations. Given the reduced number of combinations for the alga-derived communities, we decided to test all possible three-species combinations (*n* = 10).

Remarkably, despite the prevalence of biomass-reducing combinations, vessel co-culture communities displayed higher mean and top 25% biofilm biomass compared to alga-isolate communities (Vessel: MeanA_590_ = 4.2, 75th percentile A_590_ = 6.8; Alga: MeanA_590_ = 3.8, 75th percentile A_590_ = 5.2).

### Increased community complexity promoted biofilm induction

To increase community complexity, we tested three-species combinations (Table S5), building on findings from dual-species experiments. Each combination included at least one of the top biofilm-forming isolates, with some combinations containing more than one. In total, we tested 90 different combinations: 80 including vessel isolates and 10 comprising alga isolates (triple-culture vessel, TV; triple-culture alga, TA; Table S5). The predicted biofilm biomass of such multispecies combinations was calculated as the average biomass of the three corresponding monocultures. As in the co-culture assays, biofilm effects were classified as inducing, neutral, or reducing based on the same thresholds of the biofilm ratio.

Consistent with dual-species combinations, multispecies communities derived from vessel isolates displayed higher mean and top 25% biofilm biomass compared to algal communities, despite the prevalence of biomass-reducing combinations in the former (Mean_Vessel_ = 6.3, Mean_Alga_ = 4.3; 75th percentile_Vessel_ = 8.9, 75th percentile_Alga_ = 4.8). Mean and top 25% biofilm biomass also increased compared to co-culture communities.

**Fig 5:**
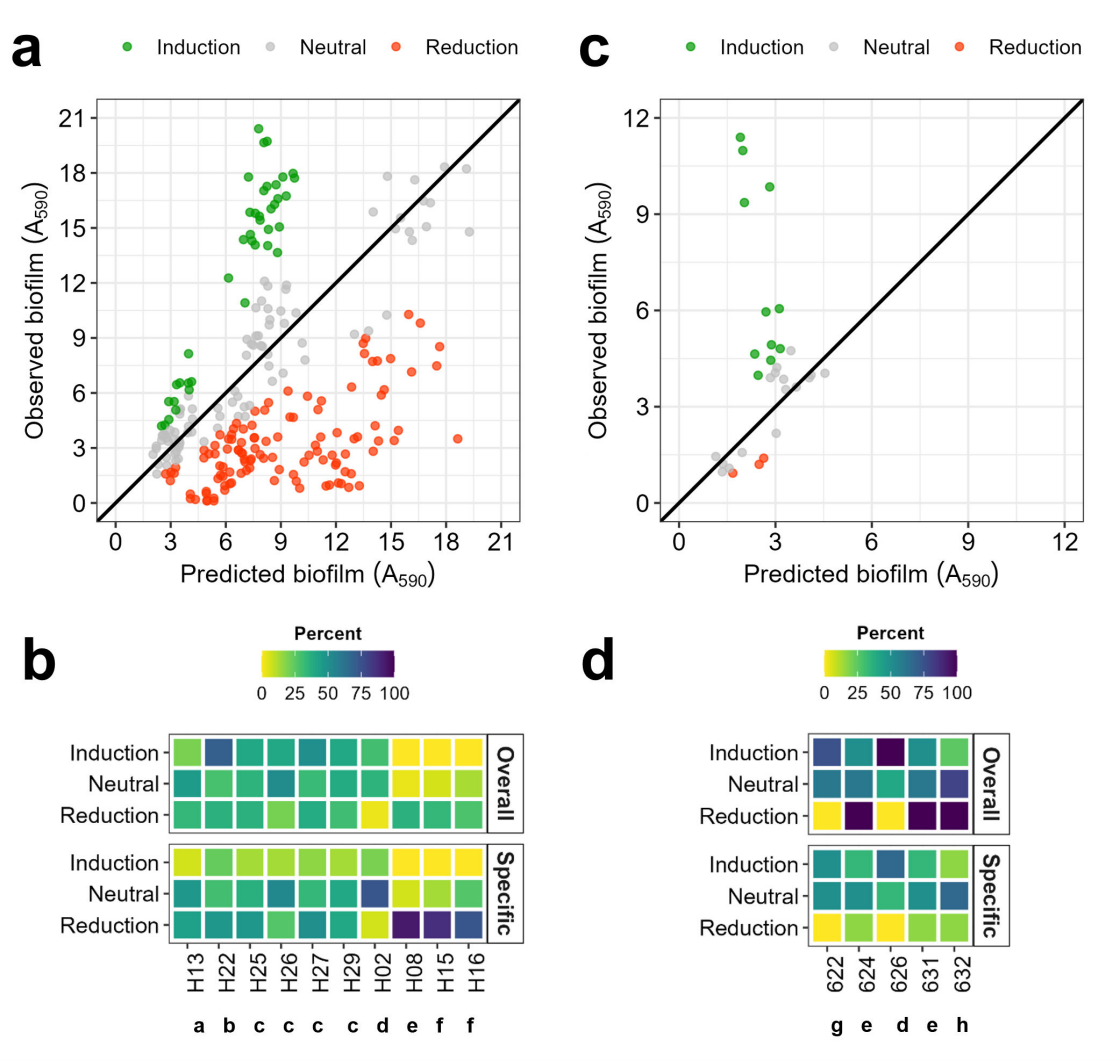
Effect of multispecies communities and their members on biofilm biomass. Biofilm formation (A_590_) was determined using a crystal violet assay after 24 h incubation in VNSS medium and 120 rpm orbital shaking. Top panels (**a and c**) represent biofilm induction/reduction of triple-culture species mixtures (y axis, Observed) compared to the average of their monospecies (x axis, Predicted). Each point represents a combination. The diagonal line indicates the threshold of induction (multispecies-monoculture ratio = 1); combinations above the line indicate induction and those below reduction. Combinations showing biofilm induction > 1.5-fold (compared to the average of their monospecies biofilms) are shown in green, while reduction > 1.5-fold is shown in red, and neutral combinations are depicted as gray points. Each point represents a combination. (**a**) Multispecies vessel-isolate communities, n = 80 combinations, three biological replicates. (**c**) Multispecies alga-isolate communities, *n* = 10 combinations, three biological replicates. Bottom panels (**b and d**) are heatmaps illustrating how frequently specific isolates were present in co-cultures of induced, neutral, or reduced biofilm formation, respectively (shown in percentages). Dissimilar letters indicated different genera. a: *Marinobacter*, b: *Maribacter*, c: *Psychrobacter*, d: *Alteromonas*, e: *Pseudoalteromonas*, f: *Paraglaecicola*, g: *Phaeobacter,* and h: *Enterovibrio*. Overall: overall presence frequency (percentage) of the strain per type of effect, calculated as the number of combinations where the strain is present in a co-culture of a specific effect/ number of combinations of that species (in percentage). Specific: effect-specific presence of the strain, calculated as the number of combinations where the strain is present in a co-culture of a specific effect/ number of combinations with that effect (in percentage). The color scale indicates the percentage of strain related to presence in multispecies with a specific outcome (induction, neutral, or reduction), where yellow color denotes less presence in biofilm-induced/-reduced combinations, while dark blue indicates higher presence. (**b**) Vessel-isolate communities, n _reduction_ = 115, n _induction_ = 40, and n _neutral_ = 85. (**d**) Alga-isolate communities, n _reduction_ = 3, n _induction_ = 12, and n _neutral_ = 15.

Multispecies communities derived from vessel isolates (TV) showed percentages of biomass induction/reduction similar to dual-species mixtures (Fig. 5a). Communities resulting in biomass reduction accounted for 47.9%, while those inducing biofilm biomass constituted 16.7% (vs 47.1% and 15% in co-culture, respectively). When analyzing how often isolates were present in induced and reduced co-cultures, H08, H15, and H16 were consistently present in combinations that reduced biofilm biomass (Fig. 5b), consistent with the results from co-culture biofilm communities. Therefore, these three isolates were excluded from further testing to maintain community stability. In contrast, 10 communities showed increased biofilm biomass compared to the sum of their biomass in monoculture (Table S5). Isolate presence in these communities differed, and there was no clear pattern for the top biofilm isolates in biofilm induction frequency, except for H22 (*Maribacter* sp.) and H27 (*Psychrobacter* sp.). These two isolates induced biofilm biomass in 70% and 50% of the communities they grew in, respectively (Fig. 5b). Of the 10 communities showing induced biomass, 4/10 contained both H22 and H27, suggesting that these strains are good partners for biofilm induction. Pairs containing H22 and other *Psychrobacter* isolates (H25, H26, and H29) were also frequent within the inducing communities, implying that interactions between *Maribacter* and *Psychrobacter* spp. are desirable for biomass production. From the biofilm-inducing communities, four were chosen for further testing at different temperatures. TV03 and TV32 both contained H22 and H27 but differed from their third partner. Moreover, we chose TV31, containing H22 and two other *Psychrobacter* (H25 and H26). For diversity, TV27 was chosen since it did not contain either H22 or H27 but three different taxa: *Marinobacter*, *Psychrobacter,* and *Alteromonas*.

When algal isolates were mixed in three-species combinations (TA), biofilm induction accounted for 37% of all combinations, while only 10% of all combinations resulted in reduction (Fig. 5c). These results are particularly interesting since multispecies combinations resulted in less biofilm reduction (−18%) and more biofilm induction (+6%). Four multispecies combinations reproducibly induced biofilm biomass: TA01, TA04, TA05, and TA07 (Table S5). All four contained 626 (*Alteromonas* sp.); whenever 626 was present in the combination, biofilm biomass was induced in multispecies communities, consistent with dual-culture experiments (Fig. 5d). The first three also contained 622 (*Phaeobacter* sp.), which seemed a good partner for biofilm induction, while TA07 contained 624 and 631, both *Pseudoalteromonas* isolates. Interestingly, both *Pseudoalteromonas* combined with 632 (*Enterovibrio* sp.) resulted in biofilm reduction. Consequently, we chose those four communities containing alga isolates for further testing.

### Multispecies biofilm communities containing vessel isolates were more robust under temperature variation

Biofilm production by eight multispecies communities was tested under different temperature conditions to investigate whether incubation temperature and/or time influenced biofilm formation. The communities TV3, TV27, TV31, TV32, TA1, TA4, TA5 and TA7 (TV = triple-culture vessel; TA = triple-culture alga; Table S5) were selected based on their biofilm biomass and reproducibility in independent experiments described above. We incubated these multispecies combinations at 4, 10 and 24°C and quantified biofilm biomass after 48 and 72 h.

**Fig 6:**
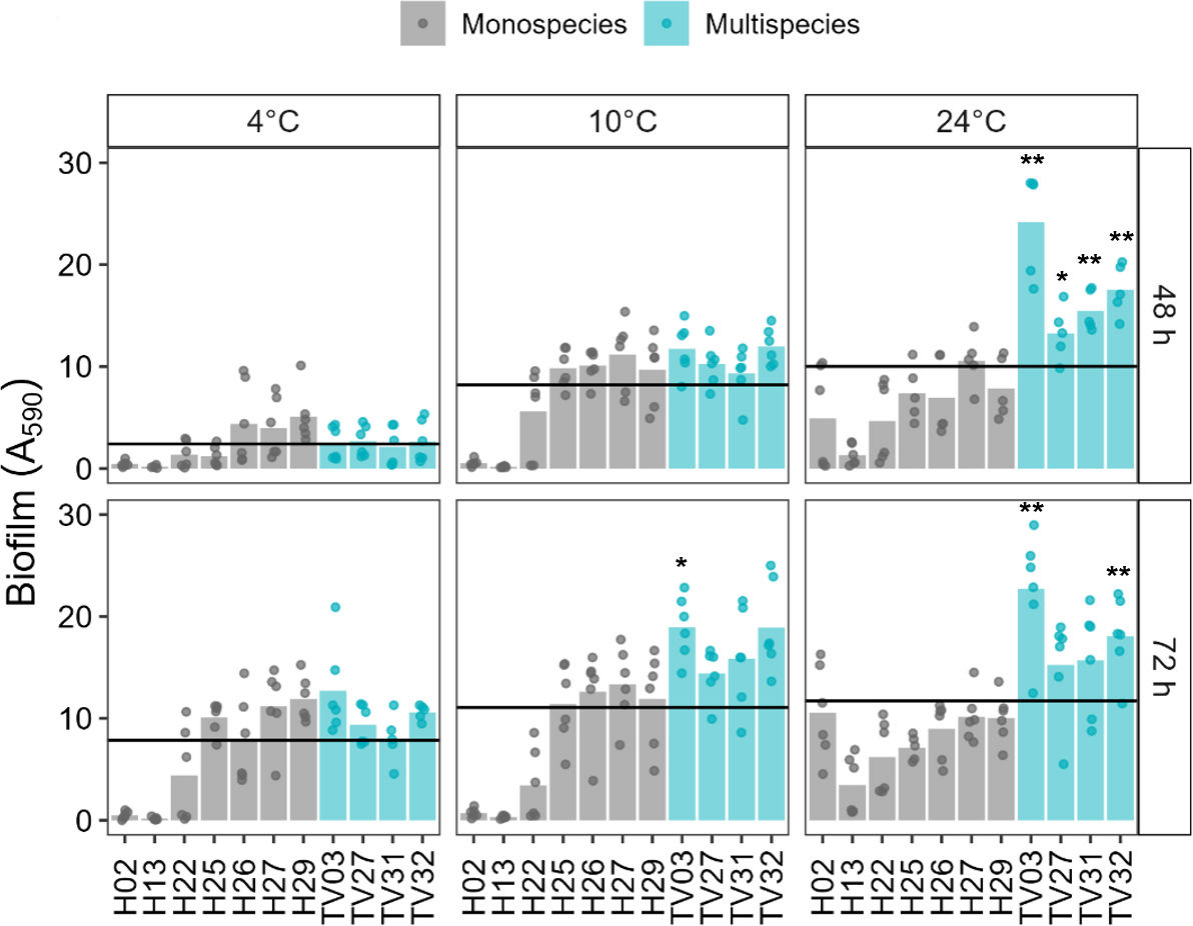
Temperature and incubation effect on adhesion of vessel multispecies communities and monospecies controls. TV: Triple-culture vessel; TV03 = H13+H22+H27; TV27 = H13+H29+H2; TV31 = H22+H25+H26; TV32 = H22+H25+H27. Bar plots indicate average biofilm biomass (A_590_) per community (blue)/ isolate (gray) incubated at 4, 10, or 24°C for 48 or 72 h. Individual points represent a biological replicate (*n* ≥ 5), each average of six technical replicates. Black crossbars indicate mean value per temperature and incubation (per facet). Asterisks denote significant *P*-values of a Mann-Whitney test comparing multispecies to the best monospecies in such mixture (Table S6). *: *P* < 0.05; **: *P* < 0.01. Black asterisks indicate significantly greater biofilm biomass in multispecies compared to monoculture.

We evaluated the effects of incubation time, temperature, and culture condition (monospecies vs multispecies) on biofilm biomass for both vessel- and alga-derived communities (Fig. 6). When considering incubation time as the main factor, longer incubation significantly increased biofilm biomass in vessel communities (*P* = 2.38×10^−04^), but not in alga communities (*P* = 0.118) (Table S6).

Similar tendencies were observed for the culture condition. Multispecies vs monospecies had a significant impact on biofilm formation in vessel communities, with multispecies communities producing more biomass than monocultures (*P* < 2.2x10^-16,^ Wilcoxon rank sum test; Table S6), while no significant difference was observed for alga samples (*P* < 0.1164, Wilcoxon rank sum test; Table S6).

Temperature significantly influenced biofilm biomass in both community types, regardless of their origin. Comparison within temperature conditions evidenced that longer incubation did not result in significantly more biomass at 24°C for vessel communities or at 4°C for alga communities. This suggested that productivity at those temperatures was limited by other factors (Table S6). In vessel communities, the culture condition continued to influence productivity across all temperatures, with more pronounced differences as the temperature increased. In contrast, for alga communities, the culture condition had a significant effect only at 10°C (Table S6).

We then compared the eight multispecies communities to their monocultures within incubation time and temperature to identify communities that displayed robust temperature-dependent biomass induction (Fig. 6). Robust biofilm biomass was considered consistent when reproducible induction of biofilm biomass in biofilm communities was observed across different temperatures. All vessel multispecies communities showed higher biofilm biomass than the average for the population, except for 4°C and 48 h incubation (Fig. 6a). However, multispecies communities were only significantly different from their best monoculture at 24°C and 48 h incubation (Table S7). Only one community was consistently and significantly more productive in terms of biofilm biomass than its monoculture constituent, TV03, suggesting high community-induced biofilm biomass within a 14°C range but also with prolonged incubation.

In contrast, multispecies communities composed of alga isolates did not show biofilm induction compared to their monospecies counterparts under any condition (Fig. S1). Isolate 626 proved highly stable regarding biofilm biomass at all temperatures and produced significantly more biomass than any of the multispecies communities in most conditions. Since all communities contained this isolate, these results may suggest dominance of 626 over the rest of the isolates.

To summarize, multispecies communities containing vessel isolates showed induced biofilm formation compared to monospecies communities at 10 and 24°C but also seemed highly robust under the temperatures tested, especially TV03 (H13 +H22+H27). Finally, temperature resilience assays also evidenced that environmental factors, and not only biological ones, can greatly impact productivity and stability of synthetic communities.

### Specific vessel isolates prevented barnacle settlement *in vitro*

Different isolates from vessel hulls displayed robust adhesion to microtiter plates and were consequently used for community assembly, as shown in Fig. 3b. We aimed to determine whether the biofilms formed by these species/communities could be sufficiently robust to protect a surface against barnacle settlement. To do this, we assessed the effect of 48 hour biofilms on settlement of *A. improvisus* cyprid larvae. *A. improvisus* is commonly used as a model barnacle species for larval settlement ecology studies because it has a wide distribution, presents a major fouling challenge to the maritime industry and settlement of the cyprid larvae can be easily observed in the laboratory (73).

**Fig 7:**
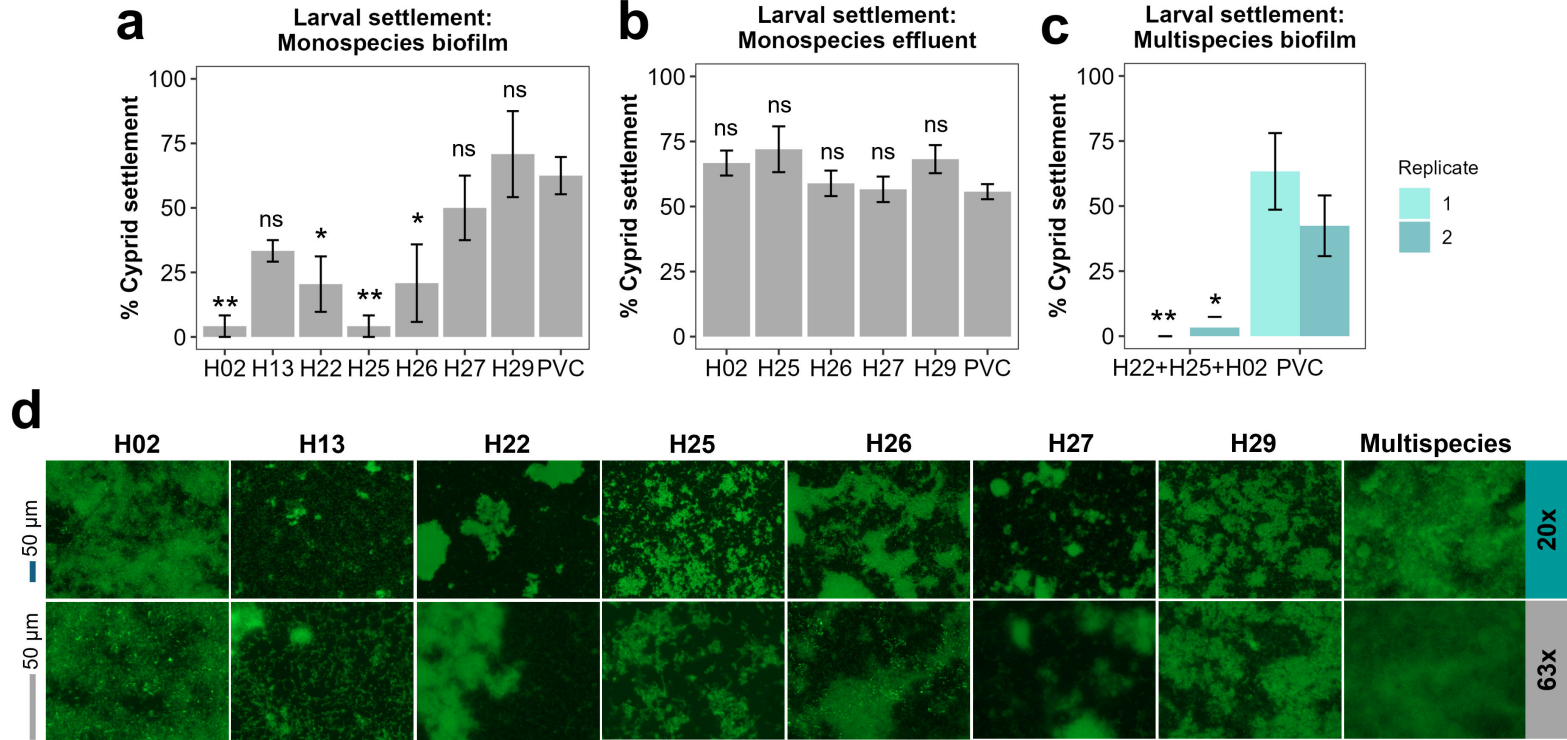
Cyprid settlement on marine isolates or communities after 48 h. Barnacle cyprid larvae of *A. improvisus* were tested for settlement on different biofilms for 48 h. Graphs show cyprid settlement percentage on 48 h bacterial monospecies biofilms on PVC coverslips (**a**), in bacterial effluent solutions (**b**), and on a multispecies biofilm composed of isolates H22+H25+H02 (**c**). Clean (no inoculated bacteria) PVC was included as a standard surface to confirm that cyprid settlement occurred within the normal range. Graphs denote mean values of six biological replicates and the standard error of the mean. The settlement percentage was calculated by expressing those settled as a proportion of the total initially added and averaging these values over the six replicates. Asterisks indicate significant difference between PVC control and isolate biofilm/community biofilm or effluent biofilm (* *P* < 0.05; ** *P* < 0.01), while ”ns" indicates no significant difference. For a and b panels, a Dunnett’s multiple comparison test was applied, given that multiple isolates were tested, while for the c panel, a Holm-Sidak method was used (*P* < 0.05). (**d**) Epifluorescence images of 48 h single- and multispecies biofilms (Multi) stained with SYTO9. Images were acquired with a Zeiss Axio Observer Z1 epifluorescence microscope with Zeiss LD-NEOFLUAR 20 x objective (20 x, left) and Zeiss N-ACHROPLAN 63x (63x, right) objectives. Scale bars represent 50 µm. Images shown are representative of three biological replicates per sample.

First, biofilms formed by individual bacterial taxa were tested in high-throughput cyprid settlement assays (data not shown). Based on the results of these screening assays and community-building experiments described in previous sections, several strain isolates and one community were identified for further use. Figure 7a presents data from monospecies biofilms, showing significant variability in cyprid settlement in response to those biofilms. To determine if soluble cues were influencing cyprid settlement, we tested the liquid medium from wells containing established biofilms. These effluent assays were conducted in clean wells. Results from the effluent assay are presented in Fig. 7b. While notable differences in cyprid settlement were observed between certain monospecies biofilms and the PVC control (Fig. 7a), differences in the settlement response to the liquid media were negligible (Fig. 7b) and we therefore concluded that any effect on larval settlement was a consequence of the biofilm bulk, rather than soluble components.

Cyprid settlement was significantly reduced on monospecies biofilms of isolates H02 (*Alteromonas* sp.), H22 (*Maribacter* sp.), H25, and H26 (*Psychrobacter* sp.) compared to the PVC reference surface. Among those, isolate H02 exhibited the most pronounced effect of inhibiting larval settlement. *A. improvisus* cyprids attempted wide-searching behavior on the H02 biofilm, an exploratory movement pattern aimed at covering a broad area to maximize the chances of encountering positive stimuli (74). However, on these biofilms, cyprids showed limited progress in such movements compared to a clean PVC coverslip (Movies S1 and S2). Interestingly, even though isolates H25–H29 were identified as *Psychrobacter* spp., only H25 and H26 significantly reduced cyprid settlement. To assess whether differences in larval settlement may be related to the properties of the bacterial biofilms, we performed imaging of their respective biofilms via epifluorescence microscopy (Fig. 7d). The purpose of imaging was to qualitatively evaluate biofilm morphology—specifically the degree of surface coverage and biofilm structure. Low-magnification images (20 x, Fig. 7d) provided an overview of colonization patterns, revealing notable differences among samples. Among the single isolates, H02 tended to form smoother, more confluent biofilms, while H22 and H25 produced more patchy or discontinuous coverage, especially when visualized at higher magnification (63 x, Fig. 7d). All images shown are representative of consistent morphologies observed across biological replicates, supporting the reproducibility of the observed patterns.

Based on the single-species results, we assembled a multispecies community composed of those isolates that exhibited the highest settlement reduction as monospecific biofilms (H02, H22, and H25) and reassessed cyprid settlement. The results of two independent experiments are shown in Fig. 7c, where settlement was reduced practically to zero. This community reproducibly reduced cyprid settlement over 48 h (Fig. 7b), achieving even greater settlement prevention than monospecies biofilms of isolates H02 and H25. We also imaged such multispecies biofilms, searching for potential morphological differences between single- and multispecies biofilms in relation to larval settlement outcomes. The multispecies biofilm exhibited more uniform and extensive surface coverage compared to the single-species biofilms, even at higher magnification (63 x, Fig. 7d). However, its morphology closely resembled that of isolate H02 alone, suggesting a dominant colonization effect of isolate H02.

Altogether, our observations indicate no direct correlation between biofilm surface coverage and the inhibition of larval settlement, given that isolates with comparable biofilm morphology showed markedly different effects on cyprid settlement (i.e., H25 vs H29 or H22 vs H27).

## DISCUSSION

Immersed surfaces are colonized by microbial communities within hours of exposure to the marine environment. From then on, the microbial communities can be instrumental in further development of benthic communities, including biofouling communities (19, 27, 75). The interactions between bacteria and macrofouling organisms are complex, dynamic and poorly understood, with some microbes inhibiting the settlement of specific macrofouling organisms, some promoting larval settlement of specific taxa, while many macrofoulers are also capable of settling on surfaces completely independently of biofilms contrary to the prevailing model of biofouling community succession (29, 76). In the field of material science, there is growing interest in biomimetic materials that can self-replicate, heal upon encountering damage, and sense and adapt to environmental change (recently reviewed by Wang et al. [77]). Microbial biofilms are of specific interest because their EPS can be considered a natural protective coating, having evolved to protect the biofilm inhabitants from environmental stressors, among other functions (78). Biofilms are ubiquitous on marine surfaces and it is therefore logical to consider whether they could be harnessed as living coatings and optimized to prevent subsequent colonization by macroorganisms—i.e. biofouling.

Previously, studies have demonstrated the potential of bacterial biofilms to protect marine living surfaces from fouling (25, 79–81), however, often with *Alteromonas*, *Pseudoalteromonas*, *Vibrio,* and *Bacillus* as dominant bacterial groups. Aguila-Ramírez et al. also reported some *Psychrobacter* strains with antifouling potential, although against other bacteria and microalgae (82). Another approach could be to use genetic modification and synthetic/engineering biology toolkits to produce bacterial strains that actively repel a wide range of macrofouling taxa. This approach has two primary drawbacks. First, GMO release into the environment is undesirable and, in many locations, entirely prohibited. Ocean vessels must have freedom of movement between territorial waters, so any novel materials that come with restrictions are unlikely to succeed in the marketplace. Of course, many existing antifouling biocides already have such restrictions, while others are under consideration. The challenges linked to the regulation of “active” strategies, not limited to traditional biocidal action, are the second reason why GMO-based approaches are avoided. Bacterial strains that actively repel larval/spore settlement through release of noxious compounds, whether GMO-based or naturally occurring, would require consideration under biocidal product regulations. Our approach to this challenge is therefore fundamentally different from those applied before. Rather than seeking active deterrence using single bacterial strains, with or without modification at the genetic level, we focus on manipulating communities of naturally occurring bacterial taxa, including taxa never previously investigated or investigated and rejected due to those individual taxa failing to show active antifouling mechanisms (e.g., *Maribacter* and *Psychrobacter* sp). Our approach avoids the use of GMOs and targets an entirely inert fouling-control mechanism, preventing surface colonization through formation of a robust protective coating (the biofilm) that prevents access of larvae to the underlying surface. The results of this study prove this concept, outlining a discovery pipeline from collection of relevant wild-type isolates, rational down-selection based on traits, and bottom-up assembly of communities with desirable characteristics. The output of this proof-of-concept study was a biofilm that prevented the settlement of barnacle larvae, by purely physical means, in short-term laboratory experiments.

Underpinning this key result, we made several observations that give an important context for further development of the community assembly approach:

First, while biofilms composed of single species may perform the necessary function, their resistance to environmental stressors will be lower than that of multispecies biofilms presenting the same phenotype and mechanism of action. We demonstrated this by exposing single and multispecies biofilms to different thermal regimes over extended periods. Such assays showed higher biofilm production of the vessel communities compared to the alga ones (Table S6). Specifically, the vessel-derived multispecies community TV03 demonstrated stable biofilm biomass production across a temperature span of 10 to 24°C compared to its individual members, proving robustness (Fig. 5a; Table S7). In contrast, none of the alga-derived communities showed similar emergent properties, while isolate 626 (*Pseudoalteromonas* sp.) displayed high tolerance under different temperatures (Fig. 5b). These findings underscore the benefit of introducing selective pressures early in the rational design of microbial communities to identify robust, high-performing consortia.

Second, the assembly pipeline proved essential as community-level biofilm production often could not be predicted from the behavior of individual isolates. Several multispecies combinations—both vessel- and algae-derived—exhibited enhanced biofilm biomass beyond what would be expected from monocultures, indicating emergent properties and enhanced, biofilm-mediated protection of community members (Fig. 5; Table S5). Notably, while no consistent pattern of isolate presence was found across all inducing communities, specific taxa like *Maribacter* (H22) and *Psychrobacter* (H27) frequently co-occurred in high-performing consortia, suggesting synergistic, biofilm-inducing interactions. Similarly, in algal-derived communities, the presence of *Alteromonas* sp. (626) was consistently associated with biofilm induction, particularly when paired with *Phaeobacter* (622). Interestingly, altering even a single member in otherwise similar consortia (e.g., replacing 622 with 632) led to markedly different biofilm outcomes (Fig. 4d and 5d; Table S5). We also found that isolates of the same genus may display interactions of distinct nature. For instance, different *Pseudoalteromonas* isolates either promoted or reduced biofilm biomass when co-cultured with other isolates (Fig. 4b). These findings support the idea that microbial interactions within designed communities are sensitive to specific isolate-level compositions. Moreover, they reinforce the unpredictability and context-dependence of microbial interactions and emphasize the value of empirical screening and bottom-up community assembly to uncover functionally robust communities.

Third, the proposed mechanism of action of presenting a physical barrier to larval settlement was vindicated by our laboratory experiments. Biofilms of vessel isolates H02, H22, H25 and H26, and a community composed of the first three, resulted in a significant reduction in settlement of cyprid larvae of the common fouling barnacle *A. improvisus* (32) (Fig. 7a and c). These isolates belonged to genera *Alteromonas* sp. (H02), *Maribacter* (H22), or *Psychrobacter* sp. (H25, H26). The inhibitory effect on cyprid settlement seemed to be linked to the physical nature of the biofilm rather than soluble cues, as evidenced by complementary effluent assays, which showed no comparable deterrent effect (Fig. 7b). Imaging 48-hour biofilms (in the absence of larvae) revealed that the multispecies biofilm was denser and exhibited near-complete surface coverage compared to the more patchy and discontinuous biofilms formed by individual isolates (Fig. 7d). This extensive coverage in the multispecies treatment may reflect the early structural contribution of isolate H02, which independently also formed relatively confluent biofilms. Interestingly, some isolates that formed patchy biofilms—such as H22, H25, and H26—still exhibited strong inhibitory effects on larval settlement. This suggests that while extensive and cohesive biofilm coverage may enhance larval deterrence, biofilm morphology alone does not fully explain the observed patterns. The cyprids of acorn barnacles perform a prescribed series of movements prior to settlement, aimed at evaluating the characteristics of settlement sites across spatial scales. If this exploratory process is unsuccessful or interrupted, settlement does not occur (74). Upon first contacting a surface, a cyprid will respond by engaging in temporary adhesion or, based on unfavorable sensory information, immediately reject the surface and return to the water column. If it remains attached, the cyprid will walk in a bipedal motion, using its highly modified frontal antennules, for a period of minutes to even hours, gathering surface-specific information and refining the search area down to a specific location suitable for permanent settlement. This is known as wide-searching behavior and is followed later by close-searching and, finally, inspection behavior prior to irreversible attachment (74). At any stage, the surface can be rejected. Our observations suggested that the majority of larvae engaged in wide-searching behavior on our functional biofilms only briefly, before returning to the water column (see Movies S1 and S2). The biofilm was, therefore, sufficiently robust and homogeneous to prevent access to the surface beneath it, disrupting the exploratory behavior of barnacles and serving as a protective coating in these short-term experiments. Not only is this mechanistic understanding important to evidence the non-biocidal mechanism of action, but it also raises the possibility of using larval behavior as an assay to further optimize biofilms for fouling prevention.

Finally, future work should focus on methods to provide a shortcut to the bottom-up assembly pipeline (e.g., by generating sufficient data to build predictive models) and addressing the many practical challenges that would accompany deployment of biofilm-based coatings in the field, such as resistance to invasion by other taxa. Russel et al. examined more than 2,000 bacterial species pairs and found that antagonism was most prevalent among closely related taxa (83), which would potentially reduce biofilm biomass. Specifically, they found that taxonomic relatedness and metabolic similarity were strong predictors of antagonistic interactions, highlighting that competition tends to be highest between strains with shared metabolic capabilities. Similar results were found by Ndiaye et al. on lactic acid bacteria, mapping over 1,100 interactions (84). While increased biofilm formation has been observed when co-cultivated bacteria originate from the same natural habitat, other studies suggest that biofilm productivity may diminish with increasing species richness —likely due to heightened competition or incompatible metabolic interactions (85). Thus, balancing phylogenetic diversity and functional compatibility will be key to engineering microbial communities with desired emergent properties. Predictive community assembly frameworks, such as those that integrate metabolic modeling, machine learning, or trait-based selection, have begun to leverage these principles to forecast outcomes of multispecies interactions. Friedman et al. demonstrated that pairwise interaction data could predict the composition of three-species combinations in synthetic bacterial communities (86). Similarly, Goldford et al. showed that simple rules based on resource competition could explain assembly patterns in microbial consortia (87). However, these models often break down in more complex environments, especially when priority effects, spatial structure, or ecological drift dominate. Despite this, predictive insights may still be valuable for designing biofilm-based coatings that are both functionally robust and resistant to invasion by environmental microbes.

In summary, we have demonstrated a novel approach toward using biofilms as future fouling-control coatings, without reliance on active mechanisms and without genetic modification using, instead, community-level engineering approaches. Our multispecies biofilms, derived from wild-type taxa, isolated from living or manmade surfaces, ultimately exhibited promising phenotypic traits compared to single-species biofilms, such as tolerance to a 14-degree Celsius temperature range. More broadly, our work exemplifies how working with nature can lead to creative applications, whereby challenges such as biofilm growth on infrastructure can be transformed into solutions—such as fouling-control coatings—by the application of new discovery pipelines.

## SUPPLEMENTAL MATERIAL

Supplemental material aem.01392-25-s0001.docx Figure S1 and captions for Movies S1 and S2.

Supplemental tables aem.01392-25-s0002.xlsx Tables S1 to S7.

Movie S1 aem.01392-25-s0003.MP4 Cyprids of *A. improvisus* exploring the surface of a clean PVC coupon (no biofilm).

Movie S2 aem.01392-25-s0004.MP4 Cyprids of *A. improvisus* exploring the surface of a biofilm of the vessel isolate H2 (*A. oceanii*).

ASM does not own the copyrights to Supplemental Material that may be linked to, or accessed through, an article. The authors have granted ASM a non-exclusive, world-wide license to publish the Supplemental Material files. Please contact the corresponding author directly for reuse.
