# Supplementary material for: Directed assembly of biofilm communities for marine biofouling prevention
Source: Appl Environ Microbiol. 2025 Aug 25;91(9):e01392-25. doi: 10.1128/aem.01392-25 (PMC12442356; doi:10.1128/aem.01392-25)
Supplement: Supplemental material — Figure S1 and captions for Movies S1 and S2. [file aem.01392-25-s0001.docx]

**Supplementary material for**

**Directed assembly of biofilm communities for marine biofouling prevention**

Cristina I. Amador^1^*, Naireen Fatima^1^, Amanda Sofie Sejer Jakobsen^1^, Lorrie Macario^1^, Phillip Pichon^2^, Nick Aldred^2^ and Mette Burmølle^1^*

^1^Department of Biology, University of Copenhagen, Copenhagen, Denmark

^2^School of Life Sciences, University of Essex, Colchester, United Kingdom

*Address correspondence to:

Universitetsparken 15, 2100 Copenhagen Ø, Denmark

Cristina I. Amador

Phone: +4542680874, e-mail: chierro@bio.ku.dk

Mette Burmølle

Phone: +4540220069, e-mail: burmolle@bio.ku.dk

**Supplementary figures –** Contains Supplementary Figure 1.

**Supplementary tables -** All supplementary tables (S1-S7) are available in the file “Supplementary tables.xlsx”.

**Supplementary movies –** Contains supplementary movies 1 and 2.


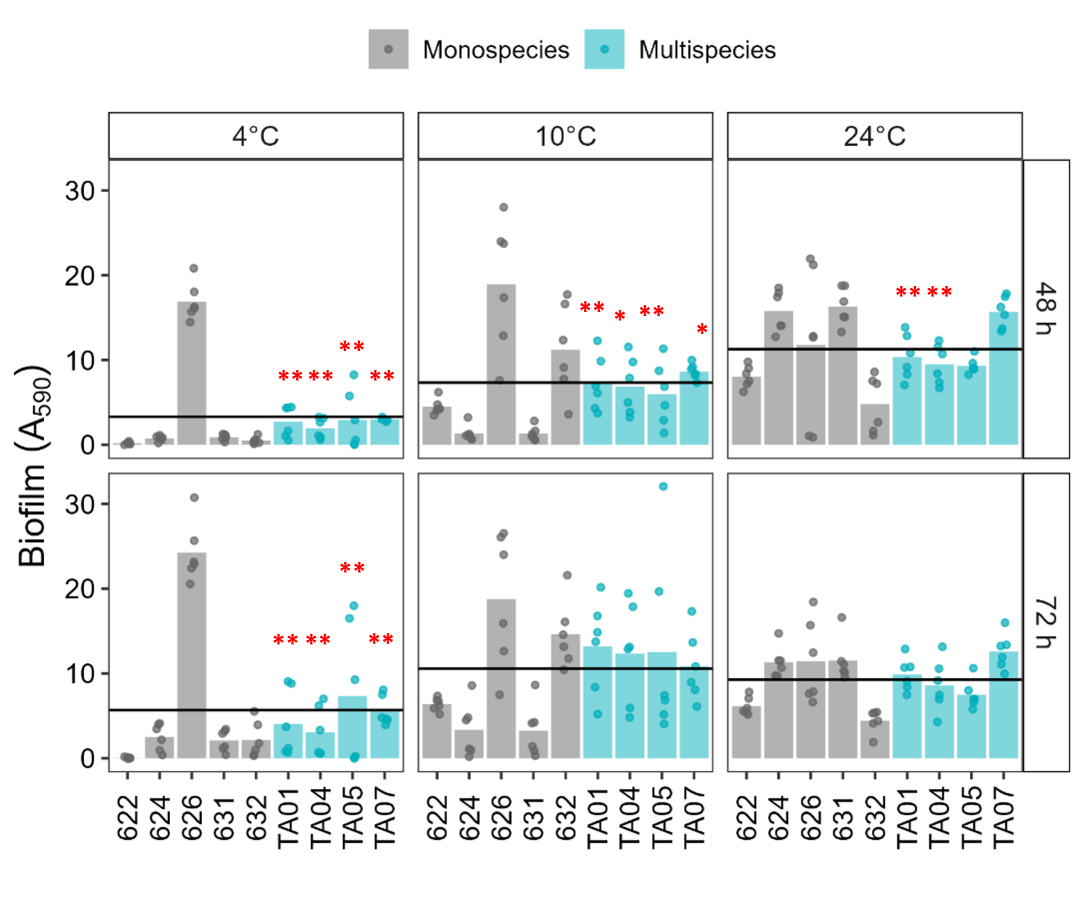


**Supplementary Figure 1. Temperature and incubation effect on adhesion of alga multispecies communities, and monospecies controls.** TA: Triple culture alga. TA01 = 622+624+626; TA04 = 622+626+631; TA05 = 622+626+632; TA07 = 624+626+631. Barplots indicate average biofilm biomass (A_590_) per community (blue)/ isolate (grey) incubated at 4, 10 or 24˚C for 48 or 72 hours. Individual points represent a biological replicate (n ≥ 5), each average of six technical replicates. Black crossbars indicate mean value per temperature and incubation (per facet). Asterisks denote significant p-values of a Mann-Whitney test comparing multispecies to the best monospecies in such mixture (Supplementary Table 6). *: *p* < 0.05; **: *p* < 0.01. Red asterisks indicate significant greater biofilm biomass in monospecies.

**Supplementary movies**

**Supplementary movie 1.** Cyprids of *A. improvisus* exploring the surface of a clean PVC coupon (no biofilm). The cyprids were cultured as per the method described for settlement assays in the method section, and then allowed to explore the surface under a Zeiss Stemi 508 stereo microscope with Axiocam colour camera. The cyprids on the clean surface are engaged in ‘inspection’ behaviour, a prelude to settlement [1].

**Supplementary movie 2.** Cyprids of *A. improvisus* exploring the surface of a biofilm of the vessel isolate H2 (*A. oceanii*).  The cyprids were cultured as per the method described for settlement assays in the method section, and then allowed to explore the surface under a Zeiss Stemi 508 stereo microscope with Axiocam colour camera. The cyprid on the biofilm is attempting exploratory movement [1] (wide search) across a broad area but making limited progress.

**References**

1. Aldred N, Alsaab A, Clare AS. Quantitative analysis of the complete larval settlement process confirms crisp’s model of surface selectivity by barnacles. *Proc R Soc B Biol Sci* 2018; **285**.
